# Emergency intubation during thrombectomy for acute ischemic stroke in patients under primary procedural sedation

**DOI:** 10.1186/s42466-021-00125-0

**Published:** 2021-05-17

**Authors:** Min Chen, Dorothea Kronsteiner, Johannes A. R. Pfaff, Simon Schieber, Martin Bendszus, Meinhard Kieser, Wolfgang Wick, Markus A. Möhlenbruch, Peter A. Ringleb, Julian Bösel, Silvia Schönenberger

**Affiliations:** 1 Department of Neurology, Heidelberg University Hospital, Im Neuenheimer Feld 400, Heidelberg, Germany; 2 Institute of Medical Biometry and Informatics, University of Heidelberg, Heidelberg, Germany; 3 Department of Neuroradiology, Heidelberg University Hospital, Heidelberg, Germany; 4 Department of Neurology, Kassel General Hospital, Kassel, Germany

**Keywords:** Thrombectomy, Acute ischemic stroke, Sedation, Emergency intubation, Emergency conversion

## Abstract

**Background:**

Emergency intubation is an inherent risk of procedural sedation regimens for endovascular treatment (EVT) of acute ischemic stroke. We aimed to characterize the subgroup of patients, who had to be emergently intubated, to identify predictors of the need for intubation and assess their outcomes.

**Methods:**

This is a retrospective analysis of the single-center study KEEP SIMPLEST, which evaluated a new in-house SOP for EVT under primary procedural sedation. We used descriptive statistics and regression models to examine predictors and functional outcome of emergently intubated patients.

**Results:**

Twenty of 160 (12.5%) patients were emergently intubated. National Institutes of Health Stroke Scale (NIHSS) on admission, premorbid modified Rankin scale (mRS), Alberta Stroke Program Early CT Score, age and side of occlusion were not associated with need for emergency intubation. Emergency intubation was associated with a lower rate of successful reperfusion (OR, 0.174; 95%-CI, 0.045 to 0.663; *p* = 0.01). Emergently intubated patients had higher in-house mortality (30% vs 6.4%; *p* = 0.001) and a lower rate of mRS 0–2 at 3 months was observed in those patients (10.5% vs 37%, *p* = 0.024).

**Conclusions:**

Emergency intubation during a primary procedural sedation regimen for EVT was associated with lower rate of successful reperfusion. Less favorable outcome was observed in the subgroup of emergently intubated patients. More research is required to find practical predictors of intubation need and to determine, whether emergency intubation is safe under strict primary procedural sedation regimens for EVT.

**Supplementary Information:**

The online version contains supplementary material available at 10.1186/s42466-021-00125-0.

## Introduction

Endovascular therapy (EVT) for patients with acute ischemic stroke due to large vessel occlusion can be performed either under general anesthesia (GA) after intubation, or in a Non-GA approach which most often means procedural sedation (PS with monitored anesthesia care) without intubation. How the type of anesthetic regimen affects the outcome of the patients is a quite controversial topic of ongoing investigations [1–4]. Reasons to choose PS are less hemodynamic impairment compared to GA, the possibility to evaluate the patients continuously during thrombectomy, avoiding ventilatory support and thus saving ICU capacity.

However, pursuing a primary regimen of PS during EVT is accompanied with the inherent risk of an emergency conversion to GA by endotracheal intubation. Patients who need deeper sedation because of agitation, who show a decline in their level of consciousness or develop respiratory insufficiency due to sedatives or due to stroke worsening, will certainly be converted to GA by most physicians in charge. In those emergency situations, endotracheal intubation has often to be performed under suboptimal conditions, when the cardiorespiratory system is already compromised, and aspiration or other complications may already have occurred. Thus, it appears preferable to avoid these situations. However, it is not well established to what extend emergency conversion influences outcome of stroke patients and how it can be predicted.

In this *post-hoc* analysis we used prospectively collected data from the observational study KEep Evaluating Protocol Simplification In Managing Periinterventional Light Sedation for Endovascular Stroke Treatment (KEEP SIMPLEST) [5], on a cohort of stroke thrombectomy patients receiving EVT primarily in PS. We aimed to identify predictors of emergent intubation and assessed different outcome parameters such as reperfusion result, early neurologic improvement, long-term functional outcome and mortality.

## Methods

### Design and main findings of the KEEP SIMPLEST study

We used collected data from the monocentric observational KEEP SIMPLEST study for this *post-hoc* analysis [5]. KEEP SIMPLEST evaluated a lean in-house periprocedural standard operating procedure (SOP) for patients with acute ischemic stroke of the anterior intracranial circulation undergoing thrombectomy in PS.

The aim of the study was to determine, whether logistical aspects and functional outcome of patients could be improved under the new SOP compared to the preceding SOP^2^. Main findings were that the new SOP was associated with reduced in-house treatment delays, while functional outcome like the National Institutes of Health Stroke Scale (NIHSS) after 24 h and modified Rankin Scale (mRS) at 3 months remained the same [5].

### Anesthetic management

All patients were primarily started on PS and monitored by neuro-intensivists (local standard). Analgosedative medication include propofol, esketamine and remifentanil. Propofol and esketamine were applied via continuous perfusor infusion and/or via bolus administration. Predefined criteria for emergency endotracheal intubation and conversion to GA were as follows: when there is an intraprocedural complication (e.g. arterial perforation), vomiting and aspiration occur, the patients were very agitated despite high doses of sedatives or when there was a compromise in airway patency or respiratory insufficiency. Eventually, the choice and dosing of sedatives as well as the decision for emergency conversion were at the discretion of the neuro-intensivist in concordance with the interventionalist.

After the intervention patients were transferred to the stroke unit when they remained under PS and to the intensive care unit when emergent intubation and GA was needed.

Emergency endotracheal intubation was performed as part of rapid sequence induction where etomidate, rocuronium and fentanyl were used as induction medication. Propofol, esketamine and remifentanil were used for maintenance of deeper sedation thereafter. During GA, mechanical ventilation aimed at avoiding hyper- or hypocapnia and hyper- or hypoxemia. A systolic blood pressure target range of 140–160 mmHg was aimed for by applying crystalloids and norepinephrine perfusor therapy in situations of hemodynamic compromise. Other details of the SOP are already published [5].

The study was approved by the local institutional review board (Ethikkommission Medizinische Fakultät Heidelberg, ID S-325/2015).

### Data collection and analysis

We obtained data from 160 patients started in PS of the KEEP SIMPLEST study from December 2016 to November 2017, where primary procedural sedation was planned for EVT of vessel occlusions in the anterior circulation. Baseline data, premorbid mRS and admission NIHSS were documented. Hemodynamic and medication data during EVT were obtained from protocol documents. Mean arterial pressure (MAP) drops of 20% were defined as intraprocedural drops of at least 20% compared to the baseline MAP on admission.

The Alberta Stroke Program Early CT Score (ASPECTS) were regularly determined in the preprocedural imaging (mostly noncontrast CT, sometimes MRI). Successful reperfusion was determined as modified Thrombolysis in cerebral infarction (mTICI) 2b-3.

Furthermore, we obtained information of whether patients where intubated before or after groin puncture for EVT. Functional outcome parameters were obtained after 24 h (NIHSS) and after 3 months (a telephone interview was conducted to assess the mRS).

### Statistical analysis

Patient and baseline characteristics are described using mean and standard deviation for continuous variables or absolute and relative frequencies for categorical variables. Differences between emergently intubated patients and non-intubated patients were assessed using the Student’s t-test for continuous variables and the Chi-squared test for categorical variables. Different outcome parameters were analyzed using regression models, where all of them include NIHSS on admission, pre mRS, and ASPECTS as covariates. The difference in NIHSS between admission and after 24 h was analyzed using a linear regression model additionally adjusted for the need of emergency intubation, a MAP drop of at least 20%, and dichotomized mTICI (2b-3 vs 0-2a). The mRS after 3 months was analyzed using an ordinal logistic regression model. Additionally, the mRS was dichotomized into unfavorable and favorable outcomes (mRS 3–6 versus 0–2) and analyzed using a logistic regression model. Both models were additionally adjusted for the need of emergency intubation, a MAP drop of at least 20%, and dichotomized mTICI. Reperfusion was assessed by mTICI, which is dichotomized as successful reperfusion 2b-3 versus failed reperfusion 0-2a, and analyzed using a logistic regression model additionally adjusted for the need of emergency intubation and a decrease in MAP of at least 20%. To evaluate patient characteristics influencing the need for an emergency intubation, a logistic regression model was used which was additionally adjusted for age. The estimated model coefficients are documented together with the corresponding 95% confidence intervals, standard errors, and *p*-values.

In the subgroup of patients who needed an emergency intubation, the time-points (before and after groin puncture) of intubation are documented using descriptive methods, as well as compared with regard to baseline characteristics using the Wilcoxon signed-rank test for continuous variables and the Chi-squared test for categorical variables. Since this is an explorative analysis, all *p*-values are of descriptive nature.

### Data availability statement

Deidentified participant data that support the findings of this study are available from the corresponding author upon reasonable request.

## Results

### Baseline characteristics of emergently intubated patients

Baseline characteristics are presented in Table 1. In total, 20 (12.5%) patients were intubated, 10 (6.25%) before (preintervention) and 10 (6.25%) after the groin puncture (intraprocedural). Reasons for intubation are listed in Table 2, the most frequent were agitation (with and without aspiration). Mean ± SD duration of the intubated state was 35.7 ± 72.2 h.

**Table 1:** Baseline and clinical characteristics

	not intubated	emergently intubated	*p*-value
*n*	140	20	
Age, mean ± SD	76.2 ± 11.08	73.3 ± 13.07	0.290
Male sex, *n* (%)	57 (40.7)	7(35.0)	0.626
Comorbidities and medication, *n* (%)			
Hypertension	117 (83.6)	13 (65.0)	0.047
Diabetes mellitus	33 (23.6)	4 (20.0)	0.723
Hypercholesterolemia	42 (30.0)	4 (20.0)	0.355
Currently smoking	21 (15.0)	3 (15.0)	1.000
Previous stroke	28 (20.0)	4 (20.0)	1.000
Coronary artery disease	33 (23.6)	8 (40.0)	0.115
Peripheral artery disease	11 (7.9)	3 (15.0)	0.290
Atrial fibrillation	47 (33.6)	7 (35.0)	0.899
Need for dialysis	1 (0.7)	0 (0.0)	0.705
Antiplatelet therapy	52 (37.1)	6 (30.0)	0.534
Oral anticoagulants	22 (15.7)	5 (25.0)	0.300
Statin therapy	37 (26.4)	7 (35.0)	0.422
NIHSS on admission, mean ± SD	13.5 ± 7.06	16.6 ± 6.29	0.068
Premorbid mRS, *n* (%)			
0–2	99 (70.7)	14 (70.0)	0.95
> 2	41 (29.3)	6 (30.0)	
ASPECTS, mean ± SD ^a^	8.2 ± 1.64	7.7 ± 2.06	0.149
Use of iv thrombolysis, *n* (%)	82 (58.6)	15 (75.0)	0.160
Site of occlusion, *n* (%)			
Left sided occlusion	67 (48.2)	12 (60.0)	0.324
M1	61 (43.6)	10 (54.5)	0.591
M2	36 (25.7)	3 (13.6)	
ICA	5 (3.6)	0 (0.0)	
ICA + M1/2	37 (26.4)	7 (31.8)	
P1	1 (0.6)	0	

^a^ 1 missing value in the group of non-intubated patients. Student’s t-test was used for continuous variables and Chi-squared test for categorical variables. *ASPECTS* Alberta Stroke Program Early CT Score, *NIHSS* National Institutes of Health Stroke Scale

**Table 2:** Reasons for emergency intubation

Reason	*n* (%)
Agitation	5 (25.0%)
Agitation + vomiting	1 (5.0%)
Agitation + aspiration	5 (25.0%)
Vomiting	4 (20.0%)
Aspiration	2 (10.0%)
Respiratory insufficiency	2 (10.0%)
Other	1 (5.0%)

NIHSS at baseline, premorbid mRS, pre-interventional ASPECTS, age, side of occlusion, sex and vascular risk factors were not substantially different between emergently intubated and non-intubated patients (Table 1).

Baseline characteristics of patients intubated before and after groin puncture did not differ substantially (Supplemental Table 1).

### Predictors for the need of emergency intubation

In a multivariable regression model including NIHSS on admission, premorbid mRS, ASPECTS, age and side of occlusion as covariables, none were associated with the need of emergency intubation (Table 3).

**Table 3:** Associations of selected baseline characteristics with the need of emergency intubation

	OR	95% CI	Std. Error	*p*-value
NIHSS on admission	1.061	0.976, 1.162	0.044	0.18
Premorbid mRS (3–6 vs 0–2)	0.996	0.288, 3.26	0.611	0.995
ASPECTS	0.878	0.649, 1.196	0.154	0.398
Age	0.974	0.931, 1.019	0.023	0.249
Left side of occlusion	0.668	0.216, 2.002	0.561	0.473

*ASPECTS* Alberta Stroke Program Early CT Score, *mRS* modified Rankin Scale, *NIHSS* National Institutes of Health Stroke Scale

### Outcome characteristics in emergently intubated patients

The duration of the EVT procedure was longer in patients who needed an intubation than in those who did not (mean ± SD; 128.8 ± 69.9 vs 88.8 ± 55.0 min; *p* = 0.004), as well as the groin-puncture to reperfusion times (mean ± SD; 107.8 ± 72.6 vs 66.9 ± 55.9 min; *p* = 0.0038). Patients who needed endotracheal intubation had higher rates of MAP drops of 20% from baseline (17 (89.5%) vs 65 (47.1%); *p* = 0.001). These MAP drops were included as covariable in a multivariable regression models with no influence on NIHSS at 24 h, mRS at 3 months or reperfusion success (Supplement Tables 2, 3, 4, 5, 6).

In terms of outcome (Table 4), emergently intubated patients showed a higher NIHSS 24 h after admission compared to the non-intubated group (mean ± SD; 19.9 ± 13.7 vs 10.4 ± 9.4; *p* < 0.001) and showed less improvement (mean ± SD; 1.4 ± 11.9 vs − 3.2 ± 9.1; *p* = 0.047). Multivariable regression models (Table 5 and Supplement Table 3) revealed that emergency intubation was not associated with less improvement in NIHSS from baseline to after 24 h (β = 4.404; 95%-CI, − 0.193 to 9.002; *p* = 0.06). There was a higher in-house mortality (Table 4) in emergently intubated patients (6 (30.0%) vs 9 (6.4%); *p* = 0.001), but the difference diminished after 3 months (7 (36.8%) vs 32 (23.2%); *p* = 0.197).

**Table 4:** Outcomes

	not intubated	emergently intubated	*p*-value
*n*	140	20	
Occurrence of at least 20% drop in MAP, n (%)	65 (47.1)	17 (89.5)	0.001
Durations			
EVT procedure, mean ± SD, minutes	88.8 ± 55.0	128.8 ± 69.9	0.004
Groin-to-reperfusion, mean ± SD, minutes	66.9 ± 55.9	107.8 ± 72.6	0.0038
Stay in ICU, mean ± SD, hours	11.8 ± 46.93	69.3 ± 75.4	< 0.001
Intubated state, mean, SD, hours		35.7 ± 72.2	
Intubated state, median, IQR, hours		8.3, 4.1–28.75	
Hospital stay, mean ± SD, hours	143.1 ± 96.7	159.2 ± 118.3	0.500
Successful reperfusion			
mTICI 2b-3, *n* (%)	124 (88.6)	14 (70.0)	0.0241
Clinical outcomes			
NIHSS at 24 h, mean ± SD	10.4 ± 9.4	19.9 ± 13.7	< 0.001
ΔNIHSS (at 24 - on admission)	−3.2 ± 9.1	1.4 ± 11.9	0.047
mRS at 3 months, *n* (%) ^a^			
0–2	51 (37.0)	2 (10.5)	0.022
> 2	87 (63.0)	17 (89.5)	
In-house mortality, *n* (%)	9 (6.4)	6 (30.0)	0.001
3 month mortality, *n* (%) ^a^	32 (23.2)	7 (36.8)	0.197
Cause of death, *n* (% of patients died)			
Cerebral	16 (50.0)	6 (85.7)	0.169
Non-cerebral	6 (18.8)	1 (14.3)	
Unclear	10 (31.2)	0 (0.0)	

^a^ missing values of 2 patients of the not intubated group and 1 in the emergently intubated group. Student’s t-test was used for continuous variables and Chi-squared test for categorical variables. *ICU* intensive care unit, *MAP* mean arterial pressure, *mRS* modified Rankin Scale, *NIHSS* National Institutes of Health Stroke Scale, *mTICI* modified thrombolysis in cerebral infarction

**Table 5:** Association of emergent intubation with functional and reperfusion outcomes

	Adjusted estimate	95% CI	Std. Error	*p*-value
ΔNIHSS (at 24 h - on admission)	4.404	−0.193, 9.002	2.327	0.06
	Adjusted OR	95% CI	Std. Error	p-value
mRS at 3 months (3–6 vs 0–2)	3.246	0.656, 24.387	0.883	0.183
mRS at 3 months (ordinal regression)	2.132	0.764, 6.098	0.527	0.151
mTICI (2b-3 vs 0-2a)	0.174	0.045,0.663	0.677	0.01

Full multivariable regression models are shown in the supplementary material. *ASPECTS* alberta stroke program early CT score, *mRS* modified Rankin Scale, *NIHSS* national institutes of health stroke scale, *mTICI* modified thrombolysis in cerebral infarction

Additionally, the rate of mRS 0–2 at 3 months (Table 4) was lower in emergently converted patients compared to no intubation (10.5% vs 37%, *P* = 0.022). However, long-term functional outcome (Table 5) was not significantly associated with emergency intubation in an adjusted regression analysis of a dichotomized mRS (0–2 vs 3–6) after 3 months (OR, 3.246; 95%-CI, 0.656 to 24.387; *p* = 0.183) or in an ordinal regression analysis of mRS shift (OR, 2.132; 95%-CI 0.764 to 6.098; *p* = 0.151).

Furthermore, successful reperfusion occurred less frequently in emergently intubated patients than in patients without intubation (14 (70.0%) vs 124 (88.6%); *p* = 0.0241). Logistic regression models after adjustment with NIHSS on admission, premorbid mRS, ASPECTS and occurrence of 20% drop in MAP, revealed an association of intubation (OR, 0.174; 95%-CI, 0.045–0.663; *p* = 0.01) with less frequent reperfusion success (Table 5).

## Discussion

GA compared to PS has been investigated in many retrospective observational studies and come out with worse morbidity and mortality in most of them [6–11]. On the other hand, three single-center RCT [1, 2, 4] comparing PS and GA and a meta-analysis (by the SAGA collaborators) [3] of patient-level data from those three RCTs showed contrary results: less disability was seen in the group, where EVT was performed under protocol-based GA than in the group with procedural sedation. This research however focused on primary, planned, at times strictly protocol-based intubation. Data are lacking in the literature to what extend emergently intubated patients, which are converted to GA differ from patients who do not need to be intubated. A relevant question is, whether it remains safe to primarily pursue a PS approach in patients at risk for emergency intubation and how to recognize the latter.

Our observed emergency intubation rate of 12.5% is comparable to the rates in the PS arms of randomized trials, such as the ANSTROKE [1] (15.6%) and SIESTA [2] (14.3%) trials. Some retrospective observational studies have yielded very low rates of 1.6% [12] or 2.5% [13] Differences in conversion rate may be due to differences in patient characteristics (i.e. stroke type and severity, anterior vs posterior circulation stroke, comorbidities), but very likely also due to differences in tolerance of cardiorespiratory and neurologic state, level of patient movement on the sides of the anesthetist or the interventionalist, institutional practice for pre-procedural patient assessment and choice of initial anesthetic technique, respectively. Thus, in studies where thresholds to intubate patients in a primarily planned fashion were lower, the rate of subsequent unplanned emergency intubation could be reduced by this factor.

### Predictors of emergency intubation

Among the baseline characteristics in our cohort, no associations of emergency intubation with NIHSS on admission, premorbid mRS, ASPECTS, age or side of occlusion was found. Additionally, in a meta-analysis of individual patient data from three single center randomized trials investigating the optimal anesthestic regimen for EVT [14], only absence of hyperlipidemia was considered to be a predictor of emergency conversion in the treatment group with primary PS. However, this was interpreted as a finding by chance, and other more biologically plausible factors like size of infarction, side of occlusion, age, severity of stroke were not associated with emergency conversion. In another retrospective analysis with data from a monocentric database consisting of 1681 patients where EVT was performed under monitored anesthesia care, only 1.6% patients needed to converted to general anesthesia. The only predictor for conversion to GA was posterior circulation stroke, while numerically higher rates was observed in patients with right sided occlusion and milder stroke.

In summary, there are no well-established predictors for patients at risk for emergency intubation during EVT in PS and it might be prudent to not rely on sole factors like stroke severity or stroke side for the decision to pursue a GA regimen primarily.

### Functional and reperfusion outcomes in emergently intubated patients

Rates of long-term favorable outcomes (mRS 0–2 after 3 month) differed significantly between emergently intubated and non-intubated patients in our study (37.0% vs 10.5%, *P* = 0.022). However, in a multivariable regression model and ordinal regression model, we could not find a significant association of emergency intubation with unfavorable outcome (mRS 3–6) or a shift in mRS. It should be noted, that some of our findings may well be a statistical consequence of very small subgroups with low power to detect a relevant effect size for long-term function after severe stroke. In the meta-analysis of the aforementioned randomized trials on GA vs PS favorable outcome (mRS 0–2) was reached in 35.1% of the patients with PS and only in 4.8% in emergently converted patients, indicating a worse long-term outcome in latter subgroup [3]. Converted patients in that cohort had a common odds ratio of 2.67 (*P* = 0.015) for a shift toward a higher mRS score than non-converted patients [14]. In the study of Noguiera et al. [12], the emergently converted subgroup had an mRS 0–2 at 90 days of 28.6% compared to 51.6% receiving monitored anesthesia care. Notably, when conversion rates in a center is very low, any possible harmful impact by emergency intubation would have a low clinical relevance.

Our findings and the available evidence show signals that emergency conversion might be associated to worse functional outcomes after EVT.

In addition, our data suggest that emergency intubation is associated with less frequent successful reperfusion during endovascular therapy (OR, 0.174; *p* = 0.01). Notably, mean groin-to-reperfusion times was longer in emergently intubated patients (66.9 vs 108.7 min, emergency intubation vs no intubation) in our cohort, which was driven by intraprocedurally intubated patients (130.1 vs 89.5 min, intraprocedural vs preprocedural). The prolongation cannot solely be attributed to the intubation procedure, hence there were possibly more challenging thrombectomy procedures (e.g. difficult access route, elongated vessels, thrombus composition) in this subgroup leading to reperfusion failure. On the other hand, in the study by Flottmann et al. [15], no significant difference was found in terms of reperfusion success between emergently converted patients and patients without conversion. While the reperfusion rates ranged between 69.7 to 79.8% in patients without general anesthesia in their study, emergently intubated patients had a successful reperfusion rate of 64%, showing a numerical difference for successful reperfusion in patients without emergency intubation. Additionally, no statically significant difference was found in the study cohort of Nogueira et al. [12], where successful reperfusion was achieved in 95.6% of patients undergoing EVT in monitored anesthesia care, while it was only achieved in 88.5% of emergently converted patients.

Albeit the differences in the other studies were not statistically significant, the data might show a trend possibly linking reperfusion failure and emergency conversion together. More studies are needed to dissect a possible relationship of reperfusion success and emergency conversion.

### Influence of hemodynamic impairment

Another concern of emergency intubation is hemodynamic impairment, i.e. hypotension, due to the need of high doses of sedatives and opioids for induction and maintenance of GA. It has been suggested to be one of the factors to render GA disadvantageous in many observational studies [16–19]. Indeed, studies focusing on BP have yielded a substantial amount of data to suggest that intraprocedural episodes of even hypotensive decrease of 10–20% before recanalization are detrimental [19, 20]. We could indeed observe a higher rate of drops MAP of 20% from baseline in the emergently intubated patients, but drops of MAP as a covariate in the multivariable regression models showed no influence on outcome.

One difference between our analysis and other studies was that we also included patients where intubation occurred before the intervention, while in other studies emergency converted to general anesthesia might have been defined as intubation during thrombectomy. Intubation before groin puncture and conversion to GA may be understood as primary GA in other places and would probably lead to exclusion of that subgroup from an analysis on emergent intubation during EVT in other studies. However, our cohort consists of patients where the primary intention was to pursue a PS strategy. Thus, patients emergently converted shortly before intervention in our setting constitute a clinically relevant subgroup, which differ from patients managed with primary GA and which should be comparable with intraprocedural emergency converters (as indicated by data shown in Supplement Tables 1 and 7).

This study has several limitations. As it was a retrospective post-hoc analysis of a single-center study, no causal relations but only associations can be derived and recommendations cannot be stated. The sample size is too small to yield robust results in long-term functional outcome. One other limitation is the fact, that some patients remained intubated and sedated at 24 h after admission (e.g. 6 of 20 emergently intubated patients were intubated longer than 24 h in our cohort, where 5 patients died after decision for transition to palliative care) and NIHSS at 24 h in these patients was obtained in the sedated state. Futhermore, inclusion of patients prior to groin-puncture could have led to additional heterogeneity. Lastly, indication for intubation was ultimately under discretion of the treating physicians and thus there might be some variability for intubation thresholds. Strengths of this study are the monocentric data source of a prospective study focused entirely on PS for EVT, the application of a protocol, and a representative mix of stroke thrombectomy candidates.

## Conclusion

We found no clinical or imaging parameter, indicating an increased risk for the need of emergency intubation. Emergently intubated patients had a prolonged intervention, reduced reperfusion success, had higher in-house mortality and a higher rate of unfavorable 3 month functional outcome. Our study signals concern regarding emergent intubation during EVT, possibly supporting lower thresholds for primary protocol-based intubation for GA, as found preferable in recent randomized trials. Our observations call for more prospective research to identify predictors of intubation need.

## Supplementary Information

**Additional file 1: Table S1.** Baseline characteristics separated in time points of intubation. **Table S2.** Multivariable regression model of NIHSS after 24 h. **Table S3**. Multivariable regression model of NIHSS Difference (NIHSS after 24 h - NIHSS at admission). **Table S4.** Multivariable regression model of mRS after 3 months (3–6 versus 0–2). **Table S5.** Multivariable ordinal regression analysis of mRS after 3 months. **Table S6**. Multivariable regression model of mTICI (2b-3 vs 0-2a). **Table S7**. Outcome parameters separated in time points of intubation.

## Data Availability

Deidentified participant data that support the findings of this study are available from the corresponding author upon reasonable request.
