# Supplementary material for: Emergency intubation during thrombectomy for acute ischemic stroke in patients under primary procedural sedation
Source: Neurol Res Pract. 2021 May 17;3:27. doi: 10.1186/s42466-021-00125-0 (PMC8130257; doi:10.1186/s42466-021-00125-0)
Supplement: Supplementary file 1 — Additional file 1: Table S1. Baseline characteristics separated in time points of intubation. Table S2. Multivariable regression model of NIHSS after 24 h. Table S3. Multivariable regression model of NIHSS Difference (NIHSS after 24 h - NIHSS at admission). Table S4. Multivariable regression model of mRS after 3 months (3–6 versus 0–2). Table S5. Multivariable ordinal regression analysis of mRS after 3 months. Table S6. Multivariable regression model of mTICI (2b-3 vs 0-2a). Table S7. Outcome parameters separated in time points of intubation. [file 42466_2021_125_MOESM1_ESM.pdf]

Supplementary data

Table S1. Baseline characteristics separated in time points of intubation

	Emergency intubation before groin puncture (preintervention)	Emergeny intubation after groin puncture (intraprocedural)	p-value
n	10	10	
Age, mean ± SD, years	74.0 ± 14.7	72.7 ± 12.0	0.677
Male sex, n (%)	4 (40.0)	3 (30.0)	0.639
Comorbidities and medication			
Hypertension, n (%)	5 (50.0)	8 (80.0)	0.160
Diabetes mellitus, n (%)	3 (30.0)	1 (10.0)	0.264
Hypercholesterolemia, n (%)	2 (20.0)	2 (20.0)	1.000
Currently smoking, n (%)	1 (10.0)	2 (20.0)	0.531
Previous stroke, n (%)	2 (20.0)	2 (20.0)	1.000
Coronary artery disease, n (%)	6 (60.0)	2 (20.0)	0.068
Peripheral artery disease, n (%)	2 (20.0)	1 (10.0)	0.531
Atrial fibrillation, n (%)	5 (50.0)	2 (20.0)	0.160
Need for dialysis, n (%)	0 (0.0)	0 (0.0)	–
Antiplatelet therapy, n (%)	4 (40.0)	2 (20.0)	0.329
Oral anticoagulants, n (%)	3 (30.0)	2 (20.0)	0.606
Statin therapy, n (%)	5 (50.0)	2 (20.0)	0.160
NIHSS at admission, mean ± SD	19.3 ± 6.2	13.8 ± 5.3	0.062
Premorbid mRS, n (%)			
0-2	6 (60.0)	8 (80.0)	0.330
>2	4 (40.0)	2 (20.0)	
ASPECTS, mean ± SD	8.3 ± 1.7	7 ± 2.26	0.176
Left sided occlusion, n (%)	8 (80.0)	4 (40.0)	0.068

The Wilcoxon signed-rank test was used for continuous variables and the Chi-squared test for categorical variables

Table S2. Multivariable regression model of NIHSS after 24 hours

	Estimate	95% CI	Std. Error	p-value
Intubation (yes vs. no)	6.406	1.829, 10.983	2.316	0.006
NIHSS at admission	0.517	0.294, 0.74	0.113	<0.001
premorbid mRS (3-6 vs. 0-2)	3.009	-0.293, 6.311	1.671	0.074
ASPECTS	-0.563	-1.419, 0.294	0.434	0.196
MAP drop of 20% (yes vs. no)	0.049	-2.868, 2.966	1.452	0.974
mTICI (2b-3 vs. 0-2a)	-8.207	-12.434, -3.981	2.139	<0.001

A linear regression model is fitted for the dependent variable NIHSS after 24h using the independent variables (covariates) Intubation (yes versus no), NIHSS at admission, premorbid mRS (3-6 versus 0-2), ASPECTS, and mTICI (dichotomized: 2b-3 versus 0-2a).

Table S3. Multivariable regression model of NIHSS Difference (NIHSS after 24h - NIHSS at admission)

	Estimate	lower 95% CI	Std. Error	p-value
Intubation (yes vs. no)	4.404	-0.193, 9.002	2. 27	0.06
NIHSS at admission	-0.48	-0.704, -0.256	0.113	<0.001
premorbid mRS (3-6 vs. 0-2)	2.968	-0.349, 6.284	1.678	0.079
ASPECTS	-0.486	-1.347, 0.374	0.435	0.266
MAP drop of 20% (yes vs. no)	0.75	-2.18, 3.679	1.483	0.614
mTICI (2b-3 vs. 0-2a)	-7.234	-11.479, -2.989	2.148	0.001

A linear regression model is fitted for the dependent variable difference between NIHSS after 24h and NIHSS at admission using the independent variables (covariates) Intubation (yes versus no), NIHSS at admission, premorbid mRS (3-6 versus 0-2), ASPECTS, and mTICI (dichotomized: 2b-3 versus 0-2a).

Table S4. Multivariable regression model of mRS after 3 months (3-6 versus 0-2)

	OR	95% CI	Std. Error	p-value
Intubation (yes vs. no)	3.246	0.656, 24.387	0.883	0.183
NIHSS at admission	1.082	1.013, 1.161	0.034	0.023
premorbid mRS (3-6 vs. 0-2)	30.102	5.817, 554.243	1.051	0.001
ASPECTS	0.742	0.556, 0.971	0.141	0.035
MAP drop of 20% (yes vs. no)	0.786	0.322, 1.894	0.449	0.593
mTICI (2b-3 vs. 0-2a)	0.076	0.004, 0.467	1.11	0.02

A logistic regression model is fitted for the dependent variable mRS after 3 months (dichotomized: 3-6 versus 0-2) using the independent variables (covariates) Intubation (yes versus no), NIHSS at admission, pre mRS (3-6 versus 0-2), ASPECTS, and mTICI (dichotomized: 2b-3 versus 0-2a).

Table S5. Multivariable ordinal regression analysis of mRS after 3 months

	OR	95% CI	Std. Error	p-value
Intubation (yes vs. no)	2.132	0.764, 6.098	0.527	0.151
NIHSS at admission	1.118	1.065, 1.175	0.025	<0.001
premorbid mRS (3-6 vs. 0-2)	9.937	4.773, 21.499	0.383	<0.001
ASPECTS	0.922	0.765, 1.108	0.094	0.39
MAP drop of 20% (yes vs. no)	1.02	0.555, 1.869	0.309	0.95
mTICI (2b-3 vs. 0-2a)	0.177	0.067, 0.441	0.477	<0.001

A ordinal logistic regression model is fitted for the dependent variable mRS after 3 months using (covariates) Intubation (yes versus no), NIHSS at admission, pre mRS (3-6 versus 0-2), ASPECTS, and mTICI (dichotomized: 2b-3 versus 0-2a). It is assumed, that the covariate coefficients are constant over all mRS values.

Table S6. Multivariable regression model of mTICI (2b-3 versus 0-2a)

	OR	95% CI	Std. Error	p-value
Intubation (yes vs. no)	0.174	0.045, 0.663	0.677	0.01
NIHSS on admission	1.083	0.998, 1.181	0.043	0.063
premorbid mRS (3-6 vs. 0-2)	0.307	0.095, 0.958	0.583	0.043
ASPECTS	0.86	0.618, 1.153	0.157	0.334
MAP drop of 20% (yes vs. no)	0.677	0.224, 1.955	0.544	0.473

A logistic regression model is fitted for the dependent variable mTICI (dichotomized: 2b-3 versus 0-2a) using the independent variables (covariates) Intubation (yes versus no), NIHSS at admission, pre mRS (3-6 versus 0-2), ASPECTS, and MAP 20% loss (yes versus no).

Table S7. Outcome parameters separated in time points of intubation

	Emergency intubation before groin puncture (preintervention)	Emergency intubation after groin puncture (intraprocedural)	p-value
n	10	10	
Occurence of at least 20 % drop in MAP, n (%) [a]	10 (100.0)	7 (77.8)	0.115
durations			
EVT procedure, mean ± SD, minutes	104.5 ± 52.2	153.0 ± 79.2	0.095
Groin-to-reperfusion, mean ± SD, minutes	85.6 ± 55.2	130.1 ± 80.6	0.167
stay in ICU, mean ± SD, hours	73.3 ± 94.9	65.3 ± 54.5	0.910
intubated state, mean ± SD, hours	46.8 ± 99.3	24.6 ± 38.6	0.364
intubated state, median, IQR, hours	13, 7.5 – 26.3	4.2, 3.5 – 32.5	0.364
hospital stay, mean ± SD, hours	110.9 ± 83.5	207.4 ± 131.9	0.059
successful reperfusion			
mTICI 2b-3, n (%)	8 (80.0)	6 (60.0)	0.329
clinical outcomes			
NIHSS at 24h, mean ± SD	19.9.3 ± 14.5	19.8 ± 13.6	0.939
ΔNIHSS (at 24h - at admission)	-0.4 ± 12.2	3.3 ± 11.9	0.427
mRS at 3 months [b] , n (%)			
0-2	2 (22.2)	0 (0.0)	0.115
>2	7 (77.8)	10 (100.0)	
inhouse mortality, n (%)	4 (40.0)	2 (20.0)	0.329
3 month mortality, n (%) [b]	4 (44.4)	8 (80.0)	0.109

[a] 1 missing value after groin puncture, [b] 1 missing value before groin puncture. The Wilcoxon signed-rank test was used for continuous variables and the Chi-squared test for categorical variables
